# The Voluntary Hospitals and the War

**Published:** 1918-09-28

**Authors:** 


### The Hospital

The Workers' Newspaper of Administrative Medicine and Institutional Life, Administration, National Insurance and Health.

No. 1686, Vol. LXIV. SATURDAY, SEPTEMBER 28, 1918. PRICE TWOPENCE

### THE VOLUNTARY HOSPITALS AND THE WAR.

Most thoughtful people, certainly all those who are interested in the maintenance and welfare of our voluntary hospitals, and who is not? especially if they have had any responsibility for their management and upkeep since the war, must be alive to many wonderful results which have been achieved. If this belief on our part is justified, we shall hope to have many communications setting forth at least one result which has accrued to the particular voluntary institution in which the reader may be most directly interested. Those of us who have shared the dangers and appraised the work, its responsibilities and stress, which most members of the staff of a hospital at the Front have to face, must have been stirred by the resulting atmosphere of these war hospitals. Who can have failed to be stimulated by the fact that every one of these workers, from the highest to the humblest, has been, and is, whole-heartedly absorbed in the claims of the sick and injured to restoration, health, and usefulness at the earliest possible date. The absence of shirkers, frivollers, and, may we say, incapables too, makes every hospital so situated a powerful agency for the development of human character at its best in all ranks of the Services. We hope that after the War every hospital in the Homeland may have caught, and may continue to inherit, both the atmosphere and devotion so characteristic of all branches of hospital work to be met with in the war zone.

We have often claimed adequate recognition for workers at Home who have, to their honour, stuck to their hospitals as a matter of duty, pushing aside the temptation to seek prominence across the water, with possibly decorations and glory. These Home-workers have done, and are rendering, services which after the war will entitle them to full recognition. They will have won and will always occupy a high position in the nursing profession as matrons, sisters, and nurses of the highest character and self-control, who have done yeoman service by eschewing laborious ease and devoting themselves whole-heartedly to the exhausting and trying work which has often fallen to the lot of many of them in Territorial and voluntary hospitals, which have provided wards for the accommodation of our fighting men when wounded, or for other cases needing continuous hospital care. It must therefore be understood that when we extol the wonderful atmosphere of our hospitals of all grades at the Front, we are fully conscious of the splendid services of the workers who have stuck to the Homeland and thereby set an example which must for all time have its steadying influence on the character of British nurses. These brave nursing sisters have afforded the strongest evidence of the strength and fibre of British women who devote their lives to the care of the sick.

We hope to bring out in successive numbers some effects of the war and their influence upon the present and future working and upkeep of our voluntary hospitals. The value of these articles must depend, in a measure, upon the cordial co-operation of our readers associated with hospitals who kindly send us the experience of the effects of the war on the particular institution with which each may be associated. The greater of the voluntary hospitals have an influence so widespread and important, and do such valuable work affecting every section of the public and many types of injury, that the people throughout the British Islands have come to take continuously, if possible, a deeper interest than ever in the work of the voluntary hospitals, one remarkable and pleasurable evidence of this being the steady growth in the contributions to their support, spontaneously given through weekly levies by the working classes, including both sexes. Those contributions, and the gifts from a much wider area of the general population to the voluntary hospitals, would be immeasurably increased if the work of propaganda was to be systematically and ably organised in every large industrial centre and great city, as well as amongst smaller communities which have the privilege of possessing a hospital in their midst. We hold that to-day is the time for every voluntary hospital to devote attention and time to the organisation of publicity, and the propaganda of the many interesting features of hospital work which are being done within its walls, not only for the immediate community which it serves, but for our warriors of all Services.

